# Translational Control of FOG-2 Expression in Cardiomyocytes by MicroRNA-130a

**DOI:** 10.1371/journal.pone.0006161

**Published:** 2009-07-07

**Authors:** Gene H. Kim, Sadhana A. Samant, Judy U. Earley, Eric C. Svensson

**Affiliations:** 1 Department of Medicine, The University of Chicago, Chicago, Illinois, United States of America; 2 Committee on Developmental Biology, The University of Chicago, Chicago, Illinois, United States of America; Victor Chang Cardiac Research Institute (VCCRI), Australia

## Abstract

MicroRNAs are increasingly being recognized as regulators of embryonic development; however, relatively few microRNAs have been identified to regulate cardiac development. FOG-2 (also known as *zfpm2*) is a transcriptional co-factor that we have previously shown is critical for cardiac development. In this report, we demonstrate that FOG-2 expression is controlled at the translational level by microRNA-130a. We identified a conserved region in the FOG-2 3′ untranslated region predicted to be a target for miR-130a. To test the functional significance of this site, we generated an expression construct containing the luciferase coding region fused with the 3′ untranslated region of FOG-2 or a mutant version lacking this microRNA binding site. When these constructs were transfected into NIH 3T3 fibroblasts (which are known to express miR-130a), we observed a 3.3-fold increase in translational efficiency when the microRNA target site was disrupted. Moreover, knockdown of miR-130a in fibroblasts resulted in a 3.6-fold increase in translational efficiency. We also demonstrate that cardiomyocytes express miR-130a and can attenuate translation of mRNAs with a FOG-2 3′ untranslated region. Finally, we generated transgenic mice with cardiomyocyte over-expression of miR-130a. In the hearts of these mice, FOG-2 protein levels were reduced by as much as 80%. Histological analysis of transgenic embryos revealed ventricular wall hypoplasia and ventricular septal defects, similar to that seen in FOG-2 deficient hearts. These results demonstrate the importance of miR-130a for the regulation of FOG-2 protein expression and suggest that miR-130a may also play a role in the regulation of cardiac development.

## Introduction

MicroRNAs (miRNAs) are ∼22 nucleotide RNA molecules that are emerging as important regulators of gene expression. First described in *C. elegans*, miRNAs have now been identified throughout the plant and animal kingdoms [1], [2], [3], [4]. These RNAs are generated from a primary RNA transcript through the sequential actions of the RNase-III enzymes Drosha and Dicer before associating with proteins of the RNA-induced silencing complex (RISC). MicroRNAs have been shown to inhibit translation or decrease mRNA stability by binding to specific sites usually in the 3′ untranslated region (UTR) of target messages, thus providing another layer of control of gene expression [5], [6], [7], [8]. While in plants most miRNAs direct cleavage of target mRNAs, most well characterized mammalian miRNA-target interactions to date have been shown to result in translational inhibition of the target [9].

Over 500 miRNAs have been cloned and sequenced, and for many, their targets are unknown. Several computational algorithms have been developed to predict miRNA target genes, but only a few of these predictions have been validated [10], [11]. Without detailed knowledge of miRNA targets, it is difficult to predict their roles in development and disease. However, given the ability of miRNAs to regulate protein translation, it is reasonable to assume that miRNAs could be involved in the regulation of these processes in vertebrates [12].

Cardiac development in mammals is a complex process that involves the coordinated activity of a number of different transcription factors and signaling molecules. For a number of these factors, it is clear that the factor's precise expression level is critical for normal development. For example, just a 60% reduction in Tbx20 protein expression during cardiac development leads to cardiac malformations [13]. Thus, even two-fold differences in critical transcriptional regulators may influence heart formation and it is therefore reasonable to suggest that miRNAs may play a role in regulating cardiac development.

Indeed, recent work has begun to elucidate the role of miRNAs in cardiac development and disease. Cardiac-restricted deletion of Dicer during early mouse embryogenesis led to embryonic lethality due to cardiac malformations, while deletion later in development led to an early post-natal lethality secondary to a dilated cardiomyopathy [14], [15]. MiR-1-2 has been shown to play a critical role in cardiac morphogenesis, as mice deficient in miR-1-2 die *in utero* of cardiac malformations [14]. Targeted deletion of miR-208, while not leading to a developmental defect, blocked the heart's ability to develop a hypertrophic response to stress [16]. These examples are likely just the beginning of the roles of miRNAs in cardiovascular development and disease that will be identified [17].

FOG-2 (also known as *zfpm2*) is transcription factor that we have previously shown to be required for cardiac development [18], [19]. In most cell and promoter contexts examined to date, FOG-2 functions as a transcriptional co-repressor by binding to the transcriptional activator GATA-4 and modulating GATA-dependent promoter activity [20], [21], [22]. Mice deficient in FOG-2 die in mid-gestation of cardiac malformations that include ventricular septal defects and ventricular wall hypoplasia [18], [19]. In this report, we describe the identification and characterization of an evolutionary conserved target site in the 3′ UTR of FOG-2 for microRNA-130a (miR-130a). When coupled to a reporter, the FOG-2 3′ UTR inhibits translation over 3-fold in a cell line expressing miR-130a. Mutation of the predicted miRNA target site within the FOG-2 UTR or blocking miR-130a relieves this translational inhibition. We show that miR-130a is expressed in cardiomyocytes and when over-expressed during embryonic development, results in a down-regulation of FOG-2 protein levels and structural heart defects similar to those seen in mice deficient in FOG-2, thus suggesting that it may play a role in regulating cardiac development.

## Results

### MicroRNA-130a is expressed in the heart and is predicted to target a conserved region of the FOG-2 UTR

**Figure 1:**
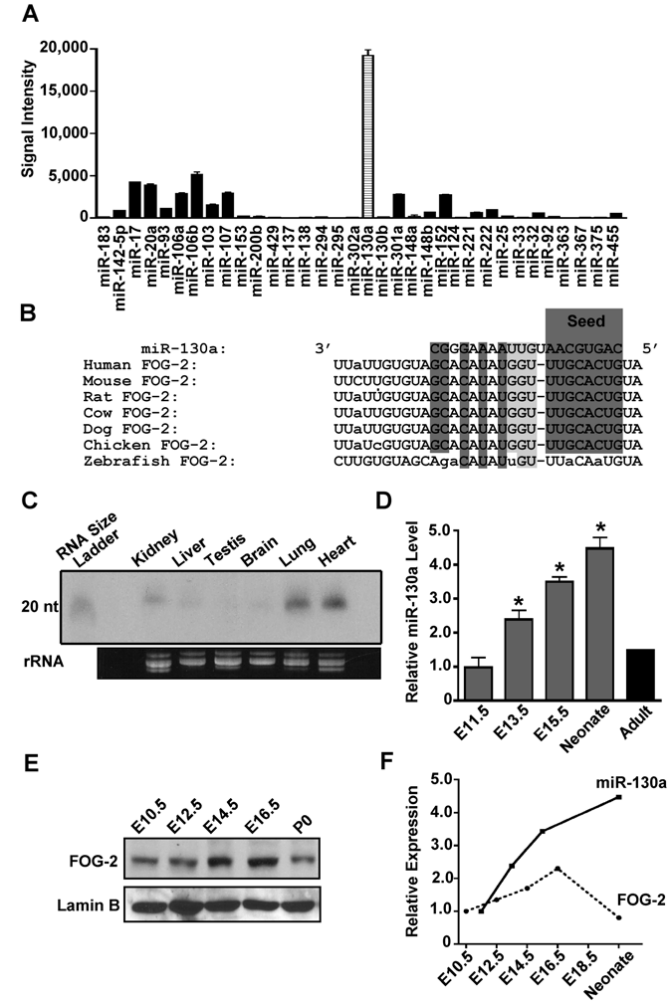
MicroRNA-130a is expressed in the heart. In (A), microarray analysis of microRNAs predicted to target the 3′ UTR of FOG-2. Signal intensity was taken as the mean of 8 probe sets and normalized to U6snRNP. In (B), an alignment of the predicted miR-130a target site in the FOG-2 3′ UTR from several different species as predicted by MicroRNA.org. Shaded boxes indicate bases pairing with miR-130a. In (C), northern analysis of 100 µg total RNA from different adult mouse tissues using a probe specific for miR-130a. Ribosomal RNA is shown as a loading control below. In (D), quantitative RT-PCR performed on RNA from pooled embryonic hearts at days 11.5, 13.5, 15.5, as well as neonatal and adult hearts. Results are normalized to GAPDH expression levels (n = 6). ‘*’ indicates a statistically significant difference (p<0.02) compared to adult. In (E), western analysis of embryonic hearts during development using an anti-FOG-2 antibody (top panel) or Lamin B (as a control for equal protein loading, bottom panel). In (F), graph of relative levels of miR-130a compared to FOG-2 protein levels during cardiac development.

Several groups have developed algorithms to predict microRNA target sites within the 3′ UTRs of vertebrate mRNAs [11], [23], [24]. One of these algorithms, PicTar (http://www.pictar.org), predicted that the entire mouse FOG-2 3′ UTR contains target sites for 34 different microRNAs. Due to the large number of miRNAs predicted to target the FOG-2 3′ UTR, we utilized a miRNA microarray to first identify those miRNAs that are highly expressed in the murine heart. An analysis of the miRNAs that are predicted to target the FOG-2 UTR demonstrated that miR-130a was the most highly expressed (Fig. 1A). Further, with the continued improvement in quality and quantity of the genomic sequences available from many diverse organisms, it was possible for us to identify FOG-2 gene homologues from distantly related species. An alignment of the FOG-2 3′ UTRs from human, mouse, rat, dog, cow, chicken, and zebrafish at the predicted miR-130a target site is shown in figure 1B. We found a high degree of sequence conservation at this site in species from human to chicken with complete identity of the “seed” sequence, critical for microRNA binding. However, this conservation in the seed sequence is lost in the zebrafish *fog2a* gene (see Discussion). Given its cardiac expression and target site conservation, miR-130a is an attractive candidate as a potential regulator of FOG-2 mRNA translation and thus is the focus of the remainder of this report.

To confirm that miR-130a was indeed expressed in the heart, we performed northern analysis on total RNA from several different adult mouse tissues using a radiolabeled probe specific to miR-130a (Fig. 1C). As can be seen, miR-130a is predominately expressed in the heart and lung, with lower amounts in the kidney. FOG-2 mRNA is predominately expressed in the heart, brain, and gonads in the adult, with lower levels in liver and lung [22]. Thus, expression of miR-130a and FOG-2 overlaps in the heart and lung and suggests that in these tissues miR-130a might modulate translation of the FOG-2 message. To examine expression of miR-130a in the developing heart, we took a PCR-based approach given the small amount of tissue available for RNA preparation at these early time points in development. Quantitative RT-PCR performed with RNA prepared from hearts at embryonic day 11.5, 13.5, 15.5, neonatal (P0), and adult revealed the highest levels of miR-130a expression at birth with levels approximately 3-fold greater compared to the adult heart (compare columns 4 & 5, Fig. 1D). This result demonstrates that miR-130a is present in the embryonic heart and regulated in a dynamic pattern throughout heart development. To determine FOG-2 protein levels during heart development, we performed western analysis using an anti-FOG-2 antibody on whole heart lysates from hearts at embryonic day 10.5, 12.5, 14.5, 16.5 and from neonatal hearts (Fig. 1E). These results reveal that FOG-2 protein levels are also dynamically regulated during heart development, with peak levels occurring at embryonic day 16.5 and diminishing in the neonate. Interestingly, as miR-130a levels peak in the neonate, FOG-2 protein levels decline (Fig. 1F). Though several factors may contribute to the dynamic pattern of FOG-2 protein expression during development, these results are consistent with the notion that miR-130a may play a role in regulating FOG-2 protein levels.

### Translational inhibition via the FOG-2 3′UTR

**Figure 2:**
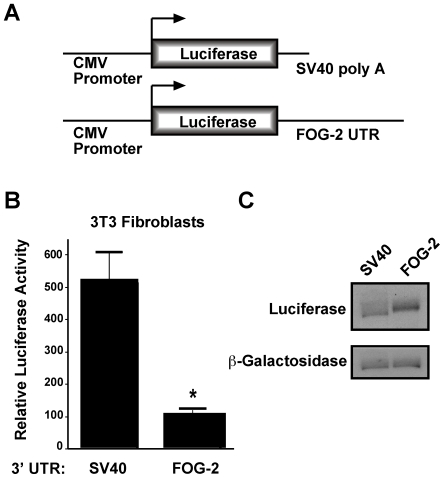
The 3′ UTR of FOG-2 inhibits mRNA translation. In (A), a schematic of the expression vector pRL and its derivative containing the FOG-2 3′UTR in place of the SV40 polyadenylation sequence. In (B), NIH 3T3 fibroblasts were transfected with the constructs shown above along with pVRβgal. Forty-eight hours post transfection, cell lysates were assayed for luciferase activity and normalized to β-galactosidase activity. Results reported are the mean±S.E.M. (n = 11). In (C), northern analysis of 20 µg total RNA from transfected fibroblasts from (B) using a probe specific to the luciferase coding region (above) or β-galactosidase (below). ‘*’ indicates a statistically significant difference (p<0.0001).

As a first step in determining the relevance of the 3′ UTR of FOG-2 for translational regulation, we generated a reporter construct in the vector pRL (Fig. 2A). The pRL vector contains the CMV promoter driving expression of an mRNA encoding luciferase and an SV40 polyadenylation signal. We generated a parallel construct in which the complete 3′ UTR of murine FOG-2 replaced the SV40 polyadenylation signal. The 3′UTR of FOG-2 contains a transcriptional terminator and polyadenylation site and thus will allow proper processing of the mRNA. We then transfected these constructs into NIH 3T3 fibroblasts, since it had been previously shown that this cell line expresses miR-130a [25]. Forty-eight hours after transfection, fibroblasts were harvested and assayed for luciferase expression. The results demonstrate that fibroblasts transfected with the FOG-2 3′ UTR construct showed a 5.2-fold lower level of luciferase activity than that of the SV40 UTR construct (Fig. 2B, p<0.0001). To determine if the decrease in luciferase activity was due to decreased message stability or translational inhibition, we performed northern analysis using the luciferase coding region as a probe. We found that luciferase mRNA levels were higher in fibroblasts transfected with FOG-2-UTR construct, indicating that the observed decrease in luciferase activity in fibroblasts transfected with the FOG-2-UTR construct was due to translational inhibition of the message rather than decreased message stability (Fig. 2C).

### MicroRNA-130a acts through the conserved site in the FOG-2 UTR to mediate translational inhibition

**Figure 3:**
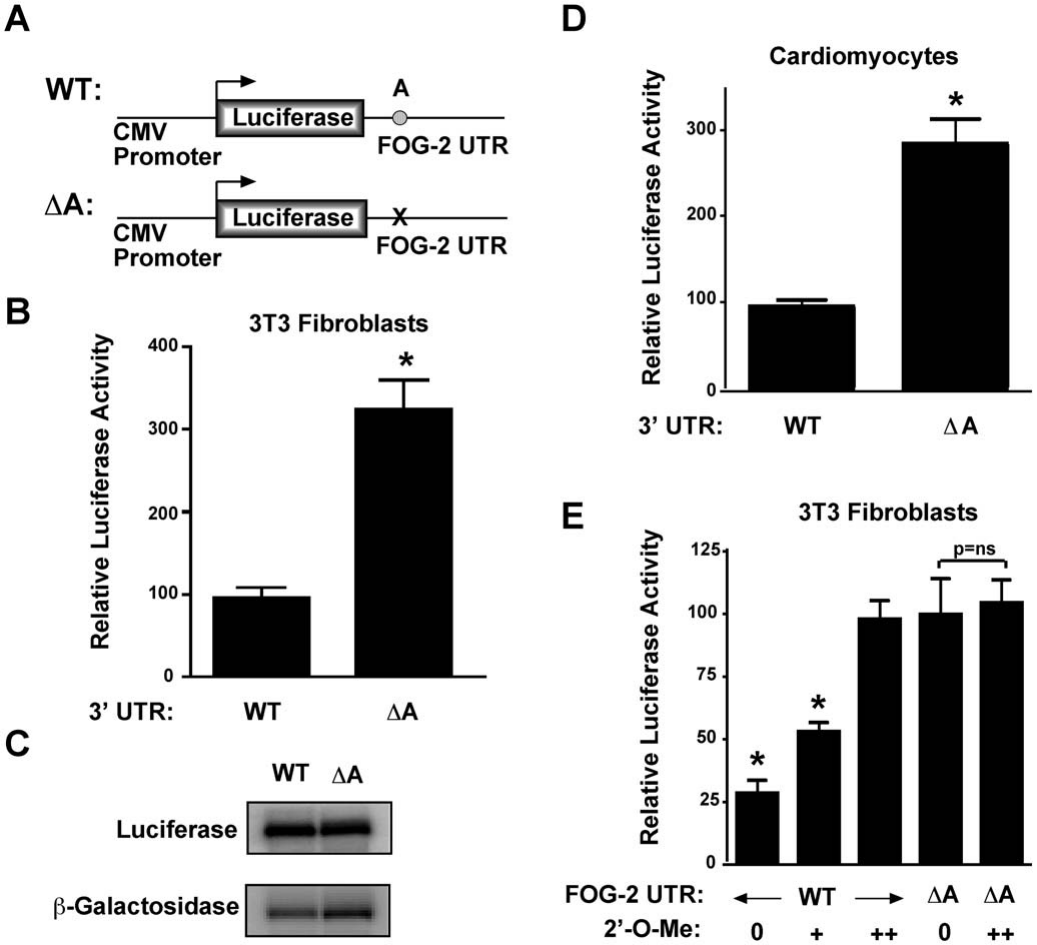
The miR-130a target site in the FOG-2 3′UTR is required for translational repression. In (A), a schematic of the constructs used to evaluate the function of the conserved region of the FOG-2 3′ UTR. In (B), NIH 3T3 fibroblasts were transfected with the constructs shown above along with pVRβgal. Forty-eight hours post transfection, cell lysates were assayed for luciferase activity and normalized to β-galactosidase activity. Results reported are the mean±S.E.M. (n = 8). In (C), northern analysis of 10 µg total RNA from transfected fibroblasts from (B) using a probe specific to the luciferase coding region (above) or β-galactosidase (below). In (D), primary neonatal cardiomyocytes were transfected with pVRβgal and a luciferase reporter containing the 3′UTR of FOG-2 or the ΔA 3′UTR mutation. Forty-eight hours after transfection, cells were assayed for luciferase and β-galactosidase activity. Results are reported as the mean normalized luciferase activity±S.E.M. (n = 20). In (E), NIH 3T3 fibroblasts were transfected with a luciferase reporter containing the 3′ UTR of FOG-2 (columns 1–3) or the ΔA mutation (columns 4 & 5) in the absence (columns 1 & 4) or presence of increasing amounts of 2′-O-methyl oligonucleotide (columns 2, 3, 5). Forty-eight hours post transfection, cell lysates were assayed for luciferase activity and normalized to β-galactosidase activity. Results reported are the mean±S.E.M. (n = 7). ‘*’ indicates a statistically significant difference (p<0.01 )

To demonstrate the importance of the putative miR-130a binding site within the FOG-2 3′UTR for mediating translational repression, we generated a reporter construct with a mutation of the predicted binding site in the FOG-2 3′ UTR (Fig. 3A). When transfected into NIH 3T3 fibroblasts, disruption of this site (ΔA) resulted in a 3.3-fold increase in luciferase activity (p<0.0001, Fig. 3B). Northern analysis confirmed that this increase was not due to an increase in mRNA stability, as message levels were identical in both samples (Fig. 3C). This result suggests that this site is required for UTR-mediated translational repression in fibroblasts. To test if this site was also inhibiting translation of FOG-2 in cardiomyocytes, we transiently transfected primary neonatal cardiomyocytes with our reporter constructs containing luciferase fused to the FOG-2 3′UTR or to the UTR with the microRNA target site disrupted (ΔA). The results shown in figure 3D indicate a 2.9-fold increase in translation upon removal of the microRNA target site (p<0.0001), suggesting the importance of this site for translational regulation of FOG-2 expression in cardiomyocytes.

To demonstrate that miR-130a is required to mediate translational repression via site A in the FOG-2 3′ UTR, we took two approaches. First, we took advantage of the ability of 2′-O-methyl-antisense oligonucleotides to inhibit specific miRNAs [26], [27]. We designed a 2′-O-methyl oligonucleotide to specifically block miR-130a and co-transfected it along with our FOG-2 3′ UTR reporter constructs into 3T3 fibroblasts. As expected, the anti-miR-130a 2′-O-methyl oligonucleotide had no effect on the translational efficiency of the reporter with a mutation of the A site (compare columns 4 & 5, Fig. 3E). In contrast, translational repression of the reporter construct was relieved in a dose-dependent fashion with the addition of increasing amounts of anti-miR-130a 2′-O-methyl oligonucleotide, resulting in luciferase levels that were equivalent to those seen with the mutated A site in the FOG-2 UTR (compare columns 3 & 4, Fig. 3E). These observations strongly suggest that miR-130a is acting through the A site of the FOG-2 3′UTR to mediate translational repression.

**Figure 4:**
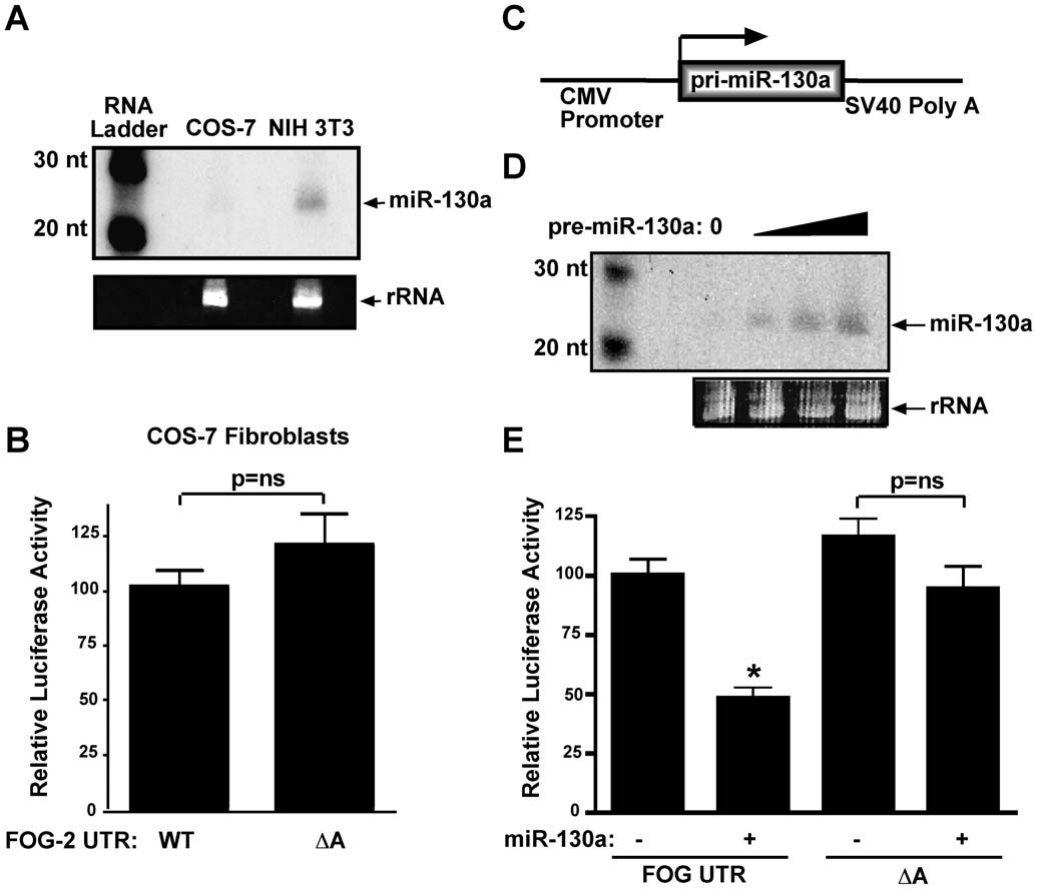
Overexpression of miR-130a inhibits translation of mRNA containing the 3′UTR of FOG-2. In (A), northern analysis using 20 µg total RNA from COS-7 or NIH 3T3 cell lines with a probe specific for miR-130a. Ribosomal RNA is shown below as a loading control. In (B), COS-7 fibroblasts were transfected with a luciferase reporter containing the 3′ UTR of FOG-2 (column 1) or the ΔA mutation (column 2) along with pVRβgal. Forty-eight hours post transfection, cells were assayed for luciferase and β-galactosidase activity. Results report the mean normalized luciferase activity±S.E.M. (n = 12). Shown in (C) is a schematic of the miR-130a expression construct. In (D), northern analysis using a probe specific for miR-130a and 20 µg total RNA from COS-7 fibroblasts transfected with increasing amounts of the miR-130a expression construct shown in (C). Ribosomal RNA is shown below as a loading control. In (E), COS-7 fibroblasts were transfected with a luciferase reporter containing the 3′ UTR of FOG-2 (columns 1 & 2) or the ΔA mutation (columns 3 & 4) in the absence (columns 1 & 3) or presence (columns 2 & 4) of the miR-130a expression construct. Forty-eight hours post transfection, cell were assayed for luciferase activity. Results reported are the mean±S.E.M. (n = 12); ‘*’ indicates statistically significant decrease in activity (p<0.001).

We also used a gain of function approach to demonstrate miR-130a's role in mediating translational repression of FOG-2. As a first step toward this end, we sought to identify a cell line that did not express miR-130a. Using northern analysis, we found that the COS-7 monkey kidney fibroblast cell line does not express detectable levels of miR-130a (Fig. 4A). We then tested our reporter constructs containing the FOG-2 3′UTR or the UTR with the A site disruption in this cell line. As can be seen in Figure 4B, the wild-type and ΔA constructs produced no significant difference in luciferase activity, consistent with the notion that miR-130a acts through the A site and that in the absence of miR-130a, deletion of this site has no effect on the ability of the UTR to modulate translational efficiency. To express miR-130a in COS-7 fibroblasts, we engineered a mammalian expression vector containing the CMV promoter driving expression of the miR-130a precursor stem loop structure (Fig. 4C). Upon expression, this precursor is recognized and processed by the miRNA processing enzymes Drosha and Dicer to produce mature miR-130a. This strategy has been used in the past to develop efficient expression vectors for other miRNAs [28], [29]. We tested the effectiveness of this strategy using northern analysis of transiently transfected COS-7 fibroblasts. As shown in figure 4D, this vector programmed expression of miR-130a in COS-7 cells in a dose-dependent fashion. To test if expression of miR-130a would repress translation of our reporter construct containing the FOG-2 3′ UTR, we co-transfected COS-7 fibroblasts with our miR-130a expression vector and our FOG-2 UTR reporter. As shown in figure 4E, the addition of miR-130a to COS-7 fibroblasts inhibited luciferase translation by 52±4% (p<0.0001). Not surprisingly, addition of miR-130a did not significantly alter luciferase activity resulting from constructs with the altered target site (ΔA, columns 3 and 4, figure 4E). Taken together with the loss of function experiments described above, these results demonstrate that miR-130a targets the FOG-2 3′ UTR to inhibit mRNA translation.

### Overexpression of microRNA-130a in embryonic cardiomyocytes results in structural heart defects

**Figure 5:**
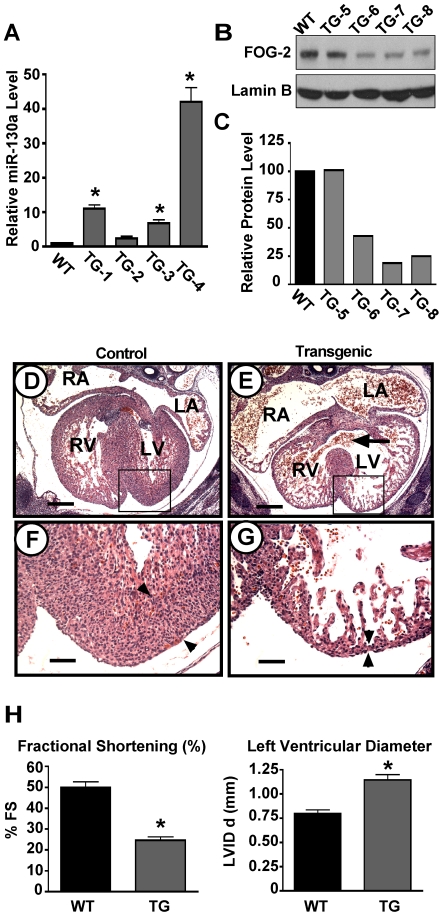
Cardiac overexpression of miR-130a results in decreased FOG-2 expression and a thin ventricular myocardial wall. In (A), expression of miR-130a as determined by quantitative RT-PCR on four wild type (WT) and four transgenic hearts (TG-1 thru 4) at embryonic day 13.5. Results represent the mean±S.E.M. of three separate experiments performed in duplicate. ‘*’ indicates statistically significant difference from wild-type, p<0.002. In (B), western analysis of wild type (WT) and transgenic hearts (TG-5 thru 8) using an anti-FOG-2 antibody. Western analysis of Lamin B was used as a control for equal protein loading. Quantitation of this blot is shown in (C), with wild type levels of FOG-2 set to 100%. In (D - G), transverse sections of embryonic day 14.5 hearts from wild type (D, F) and β-MHC-miR-130a transgenics (E, G) stained with hematoxylin and eosin. Note the ventricular septal defect (arrow, E) and the thin compact zone of ventricular myocardium (arrowheads, F compared to G) seen in the transgenic embryos. In (H), echocardiographic determination of left ventricular fractional shortening (left panel) and left ventricular end diastolic diameter (right panel) in embryonic day 14.5 transgenic and non-transgenic embryos. ‘*’ indicates a statistically significant difference (p<0.0005).

As we have shown previously, FOG-2 deficient mice die at approximately embryonic day 13.5 due to multiple cardiac defects that include ventricular septal defects and ventricular wall hypoplasia [18], [19]. It was our hypothesis that overexpression of miR-130a in the embryonic heart would lead to similar defects by inhibiting translation of FOG-2 mRNA. To study the effect of miRNA-130a on cardiac development, we generated transgenic mice with expression of the miR-130a precursor driven by the β-MHC promoter. This promoter has been previously shown to direct high-level expression in cardiomyocytes beginning at embryonic day 9 [30]. Due to the lethality of FOG-2 deficient mice at embryonic day 13.5, we suspected that transgenic over-expression of miR-130a might also lead to embryonic lethality and thus we would be unable to establish a viable transgenic line. Indeed, of 40 live born pups examined, only 2 were transgenic (5%), while the transgenic rate of embryonic day 13.5 embryos was approximately 20%, suggesting a partial embryonic lethality of transgenic embryos. Further, the 2 live born transgenic pups were found not to express increased levels of miR-130a in their hearts (data not shown), suggesting that these animals survived because the transgene had been silenced in these animals, perhaps due to integration site effects. Therefore, we chose instead to analyze our miR-130a transgenics as F0 lines between embryonic days 13.5 to 14.5 of development. As a first step, we harvested transgenic embryos at embryonic day 13.5 and isolated total RNA to perform quantitative RT-PCR to confirm increased expression of miR-130a. We found that compared to wild type littermates, 75% of βMHC-miR-130a transgenic embryos displayed a significant increase in miR-130a expression (Fig. 5A). Not surprisingly, the level of increased expression varied between transgenic embryos from 9 to 42 fold, likely due to integration site and copy number differences. Of note, one of the four transgenic embryos examined did not express significantly higher levels of miR-130a, consistent with our observation of live born transgenic pups as described above. To demonstrate the effect of miR-130a overexpression on FOG-2 protein levels, we performed western analysis of βMHC-miR-130a transgenic hearts using an anti-FOG-2 antibody. Three of four hearts examined showed significantly reduced FOG-2 protein levels (Fig. 5B). Further, quantitation of protein levels found that FOG-2 was reduced by 75 to 80% in half of the embryos examined (Figure 5C). These results support the hypothesis that translation of FOG-2 mRNA is regulated by miR-130a in cardiomyocytes *in vivo*.

As a final step in our characterization of miR-130a transgenic embryos, we performed histologic and echocardiographic analysis of transgenic hearts at embryonic day 13.5 to 14.5. At embryonic day 13.5, we observed morphologic defects in the hearts in 5 of 9 embryos examined (i.e., 9 independent lines). At embryonic day 14.5, we observed morphologic defects in 4 out of 5 transgenic embryos examined (i.e., an additional 5 independent lines). At both embryonic day 13.5 and 14.5, the left and right ventricular free wall compact zone of transgenic hearts was thin when compared to hearts of non-transgenic littermates. At embryonic day 14.5, the left ventricular wall compact zone of affected hearts was on average 2 to 3 cells thick, while that of wild-type littermates was 15 to 18 cells thick (Fig. 5F & G). To evaluate the functional consequences of the thin myocardium seen in transgenic embryos, we used fetal echocardiography (Fig. 5H). At embryonic day 14.5, transgenic embryos were found to have a reduced fractional shortening (24% vs. 50%, p<0.0001) and increased left ventricular end-diastolic diameter (1.15 mm vs. 0.79 mm, p<0.0001), demonstrating severe left ventricular dysfunction in transgenic embryos likely secondary to the thin ventricular compact zone. Histologic analysis also revealed that the developing heart valves were normal in appearance and there was no mal-alignment of the great vessels of the outflow tract. However, in 4 of the 5 transgenic hearts examined at embryonic day 14.5, a membraneous ventricular septal defect was present (arrow, Fig. 5E), while none of non-transgenic littermates examined had a ventricular septal defect. The thin compact zone and ventricular septal defect are similar to those seen in FOG-2 deficient hearts, providing further support to the notion that miR-130a may play a role in the regulation of cardiac development through the modulation of FOG-2 translation.

## Discussion

The regulation of protein translation by miRNAs is an emerging field that will likely have a significant impact on aspects of developmental biology and cancer. In this report, we have demonstrated that the 3′ UTR of FOG-2 contains an evolutionarily conserved region that regulates translational efficiency in a miR-130a-dependent fashion. While many plant messages targeted by miRNAs are cleaved and degraded, it is thought that most vertebrate miRNAs function through translational inhibition of their target mRNAs [4]. Our results are consistent with this notion, although do not rule out that miR-130a may also target other messages for degradation instead of translational inhibition as seen with the FOG-2 3′UTR. The 3.3-fold miR-130a-dependent translational repression seen on the FOG-2 3′ UTR (Fig. 3) is comparable to what has been reported for other vertebrate UTRs examined to date [11], [31], [32], [33]. MiR-143 has been shown to inhibit ERK5 mRNA translation 2-fold through a site in its 3′ UTR, miR-20a inhibits translation of the E2F1 mRNA approximately 4-fold, and miR-375 inhibits translation of Myotrophin mRNA 2-fold. The mechanism of translational repression mediated by miRNAs is currently unclear, but is an area of active research interest.

MicroRNA-130a was first cloned from mouse cerebellum and subsequently identified in mouse embryonic stem cells [25], [34]. Northern analysis has confirmed expression of miR-130a in mouse embryonic fibroblasts and both human and mouse ES cells as well as murine NIH 3T3 fibroblasts and mouse lung [25], [35], [36]. Consistent with these results, we have shown that miR-130a is expressed in a tissue-restricted fashion, with highest levels in the heart and lung. Other miRNAs have been described to have a tissue-restricted pattern of expression including miR-1, expressed in the heart and skeletal muscles, and miR-375, expressed solely in pancreatic islets [31], [34], [37]. It is likely that as more miRNAs are characterized, many will have a tissue or developmentally restricted pattern of expression.

It is possible that other miRNAs also target the FOG-2 3′UTR. Indeed, the PicTar algorithm has identified 34 different microRNAs that may target FOG-2 (see Fig. 1). It is possible that some of these microRNAs may also modulate FOG-2 translation in cardiomyocytes or in other cell types. Thus, it is likely that the combinatorial effects of many different microRNAs acting in concert on a specific UTR may provide the means to finely regulate protein levels within various cells types and at different stages of development.

As summarized in a number of excellent reviews, there is growing evidence that miRNAs play a role in regulating cardiovascular development [38], [39]. Given our observation that miR-130a expression is dynamically regulated in the heart during embryonic development, miR-130a may also play a role in regulating cardiac development, at least in part by translational regulation of FOG-2, mediated through a conserved site located in the FOG-2 3′UTR. In addition to targeting FOG-2, miR-130a also likely targets other genes involved in heart development. Thus, we cannot rule out the possibility that translational inhibition of other targets in the developing heart by miR-130a may also contribute to the phenotype we have observed in our miR-130a transgenic embryos.

As described in the Results section, only about two-thirds of our transgenic embryos have a discernable cardiac phenotype. This may be explained in part by approximately 25% of our F0 transgenic embryos not expressing significantly increased levels of miR-130a (Fig. 5A). The lack of expression of a transgene is often seen in the generation of any transgenic mouse line and is believed to be due to silencing effects of the site of transgene integration. In addition, another 25% of our transgenic embryos expressed levels of miR-130a that were sufficient to only decrease FOG-2 protein levels by less than 50%. Since mice heterozygous for a disruption in the FOG-2 gene do not have any cardiac phenotype [18] and only express 50% of normal FOG-2 levels (G. K. and E. S., unpublished observations), it is likely that transgenic mice with low level miR-130a expression may also not develop cardiac malformations due to only modest reductions in FOG-2 protein levels.

During cardiac development, FOG-2 is expressed in the myocardium, endocardium, and epicardium. Targeted disruption of the FOG-2 gene results in embryos with hyperplastic endocardial cushions, thin ventricular walls, and ventricular and atrial septal defects [18]. In the miR-130a transgenic embryos, FOG-2 levels should only be reduced in cardiomyocytes, since the β-MHC promoter used to drive expression of miR-130a in our transgenic mice is not active in the developing endocardial cushions or epicardium [30]. Thus, it is not surprising that the miR-130a transgenic embryos do not fully recapitulate the phenotype of the FOG-2^−/−^ embryos. However, the thin compact zone seen in the miR-130a transgenic mice is similar to that seen in the FOG-2 deficient mice, suggesting that this phenotype may be due to reduced FOG-2 levels. It is currently unclear how reduced cardiac FOG-2 levels lead to a thin ventricular wall, but the results presented in this report suggest that it is due to the loss of FOG-2 specifically in cardiomyocytes, rather than the endocardium or epicardium. The ventricular septal defect seen in the miR-130a transgenics may be due to the failure of the myocardium in the proximity of the developing endocardial cushions to send the appropriate signals to allow for the normal maturation of the membraneous interventricular septum.

Finally, it is interesting that the miR-130a target site in the FOG-2 UTR is highly conserved in species from chicken to human, but is not conserved in the two zebrafish orthologues of FOG-2, *fog2a* and *fog2b* [40]. The loss of conservation of this site in the zebrafish FOG-2 genes may be explained by the lack of expression of these genes in the developing heart, in contrast to their orthologues in higher vertebrates. This suggests that miR-130a may have a distinct function in higher vertebrates during heart development as compared with its function in zebrafish. A direct demonstration of miR-130a's function during development must await the generation of mice with a targeted disruption of the miR-130a gene.

## Materials and Methods

### Plasmids

pVRβGal, a plasmid that expresses the *LacZ* gene under the control of the CMV promoter, has been previously described [41]. pRL-CMV was obtained from Promega (Madison, WI). The murine FOG-2 3′ UTR (4026–4056 bp of Genbank accession number AF118845) was amplified from pcDNA-FOG-2 [22] using PCR and the primers 5′-GCGTCTAGAACTAACTGAGTTACT and 5′-GCGGATCCCCAACAATTTGGAATT. This 1009 bp fragment was cloned into the XbaI/BamHI site of pRL-CMV after the SV40 polyadenylation sequence had been removed. The ΔA construct was generated by using PCR-based mutagenesis of the 1009 bp FOG-2 UTR and the primers 5′-AGCTACTATAGTGTCCGTATCGCTAGATGCACACAAGAACTATACAATCA and 5′-GTTCTTGTGTGCATCTAGCGATACGGACACTATAGTAGCTTTTAAAGAAA. This generated a FOG-2 UTR with a 20 bp mutation in the predicted miR-130a binding site. The resulting fragment was cloned into the XbaI/BamHI site of pRL-CMV as above. The miR-130a expression construct was generated using PCR to amplify a 467 bp fragment encoding the miR-130a precursor from mouse genomic DNA with the primers 5′CACTCGAGCTCTGGACAGGTCTACAAAAATGGand 5′-CATTGCGGCCGCCCTTGAGAAGTGTCAAATGATGG. This fragment was cloned into the NotI/XhoI sites of pcDNA3 (Invitrogen, Carlsbad, CA). The transgenic miR-130a over-expression construct, pβ-MHC-miR-130a, was generated by inserting the miR-130a genomic fragment described above into a plasmid containing 5.6 kb of the murine β-MHC promoter and the human growth hormone polyadenylation signal [42]. All constructs were verified by DNA sequencing.

### Cell culture and Transfections

Primary culture of rat neonatal cardiocytes were prepared as previously described [43] and plated into rat tail collagen-coated 12-well plates. Seventy-two hours after plating, cardiocytes were transfected with 650 ng luciferase reporter plasmid, 350 ng pVRβGal, and 3 µl Fugene6 in SMEM to a total volume of 100 µl. Cells were incubated for 3 hours at 37°C, 5% CO_2_ and then 2 mls growth media was added. Cardiomyocytes were transfected in five independent experiments performed in quadruplicate. COS-7 and NIH 3T3 fibroblasts were transfected using Superfect (Qiagen, Valencia, CA) with 200 ng pVRβGal, 2 µg luciferase reporter plasmid, 0–2 µg of the anti-miR-130a 2′-O-methyl oligonucleotide (5′-GCCCUUUUAACAUUGCACUC) and pcDNA3 to a total of 5 µg DNA as described previously [44]. All fibroblast transfections were carried out in triplicate in 3 to 4 independent experiments.

### Luciferase and β-galactosidase assays

Forty-eight hours after transfection, cells were harvested and lysed in 300 µl of Renilla 1× Reporter Lysis buffer (Promega) following manufacturer's instructions. Luciferase activity was measured using 20–75 µl of this lysate with 100 µl luciferin using a TD−20/20 luminometer. β-galactosidase activity was measured in these lysates as previously described [44]. Relative luciferase activity was calculated as the raw luciferase activity divided by the β-galactosidase activity.

### Northern Analysis

Total RNA was isolated from cell lines or adult mouse tissues using the TRIZOL reagent (Invitrogen) according to the manufacturer's instructions. For northern analysis of transfected cell lines, 10 µg of total RNA was resolved on 1.2% denaturing agarose gel electrophoresis followed by transfer to a Hybond N+ membrane (Amersham Biosciences, Piscataway, NJ). The membrane was hybridized to a ^32^P-labeled fragment of the luciferase cDNA (bp 836–2005 of pRL-CMV) or β-galactosidase cDNA as described previously [22]. For northern analysis of miR-130a, 100 µg of total RNA was resolved by 15% denaturing polyacrylamide gel electrophoresis and then transferred to a Hybond N+ membrane using a semidry transfer apparatus (Bio-Rad, Hercules, CA) at 300 mA for 90 mins. Following transfer, this membrane was crosslinked and prehybridized at 35°C in Hyb buffer (50% formamide, 5×SSPE, 1% SDS, 5xDenhardt's solution, and 33 µg/ml denatured herring sperm DNA) for 1–2 hours. Subsequently it was hybridized with 20 ng/ml of a ^32^P-radiolabeled LNA oligonucleotide complementary to miR-130a( 5′-GCCCTTTTAACATTGCACTC) in Hyb buffer at 35°C overnight. The following day the membrane was washed twice for 15 mins with 2×SSC, 0.1% SDS at 35°C and then exposed to film overnight.

### MicroRNA Microarray

Total RNA was prepared from two hearts of 8-week old CD-1 mice using TRIZOL reagent (Invitrogen) according to the manufacturer's instructions. One microgram of total RNA from each preparation was labeled with Hy3 using the miRCURY LNA microRNA Power labeling kit (Exiqon) and then hybridized to miRCURY LNA microRNA array v.10.0 (Exiqon). Array scanning was performed with a GenePix 4000B scanner.

### Western Analysis

Embryonic day 13.5 hearts were harvested and 50 µg of total cell lysates were resolved by 7% SDS-PAGE followed by western transfer to a nitrocellulose membrane. The membrane was blocked with Blotto (10 mM Tris, pH 7.5, 140 mM NaCl, 0.05% Tween-20, 5% powdered milk) for 1 hour at room temperature, followed by incubation with a 1∶1000 dilution of anti-FOG-2 rabbit polyclonal antibody (Santa Cruz, M-247) or a 1∶500 dilution of anti-Lamin B antibody (Santa Cruz, M-20) in Blotto. The membrane was washed with TBST (10 mM Tris, pH 7.5, 140 mM NaCl, 0.05% Tween-20), incubated for 1 hour with a 1∶5000 dilution of goat anti-rabbit antibody conjugated to horseradish peroxidase. The blot was then washed extensively and developed using a commercially available kit (ECL-plus, GE Healthcare, Piscataway, NJ). Quantitation was performed by densitometry using a Molecular Dynamics STORM 860 Phosphoimager and intensity of FOG-2 signal was normalized to that of Lamin B to control for variations in protein loading.

### Quantitative PCR

Total RNA was isolated as previously described. 10 ng of total RNA was used to perform reverse transcription using the TaqMan® microRNA assays kit and MicroRNA Reverse Transcription kit (Applied Biosystems) according to the manufacturer's instructions. PCR amplification was then performed using TaqMan® 2X Universal PCR Master Mix (Applied Biosystems). Data analysis was performed according to the technique described by Tichopad et al. and normalized to results obtained with primers specific to GAPDH. Results represent three independent experiments performed in duplicate (n = 6) and are reported as the mean±S.E.M.

### Transgenic Mouse Generation

All mice were cared for and experiments performed in accordance with the policies of the University of Chicago Animal Care and Use committee. Pronuclear injections were performed into CD-1 or C57BL/6 oocytes using a 10 kb fragment of pβ-MHC-miR-130a containing 5.6 kb of the β-MHC promoter driving expression of miR-130a. Thirteen to fourteen and a half days following injection, embryos were harvested and genomic DNA was isolated from embryonic yolk sac. A PCR-based assay using primers specific for the β-MHC-miR130a transgene (5′-AAGATTGTGCCACTGCACTCCAGC and 5′-CCTCAGCATCACTGCATTTTCTCC) was used to identify transgenic embryos. Embryos were fixed in 10% formalin at 4°C, then dehydrated with washes of increasing concentrations of ethanol (50%, 70%, 95% and 100%). Embryos were embedded in paraffin, sectioned, and stained with hematoxylin and eosin. Images were obtained using a Zeiss Axiophot microscope.

### Echocardiography

We performed fetal echocardiography in mice using inhaled isoflurane (**∼**1%) for anesthesia, delivered via nose cone. The abdominal hair was removed from the pregnant dam with a topical depilatory agent. Body temperature was maintained using a heated imaging platform and warming lamps. The embryos were imaged *in utero* with a VisualSonics Vevo 770 machine using a 30 MHz high-frequency transducer. Two-dimensional images were recorded in approximately the parasternal long- and short-axis projections with guided M-mode recordings at the midventricular level in both views. Left ventricular internal dimensions at diastole and systole (LVIDd and LVIDs, respectively) were measured in at least three beats from each projection and averaged. Left ventricular fractional shortening [(LVIDd – LVIDs)/LVIDd] was calculated from the M-mode measurements. Relative positions of individual embryos within the uterus were noted and following echocardiography, all embryos were harvested for genotype determination as described above.
